# Supplementary figures and images for: RBP-J regulates homeostasis and function of circulating Ly6Clo monocytes
Source: eLife. 2024 Feb 26;12:RP88135. doi: 10.7554/eLife.88135 (PMC10942619; doi:10.7554/eLife.88135)

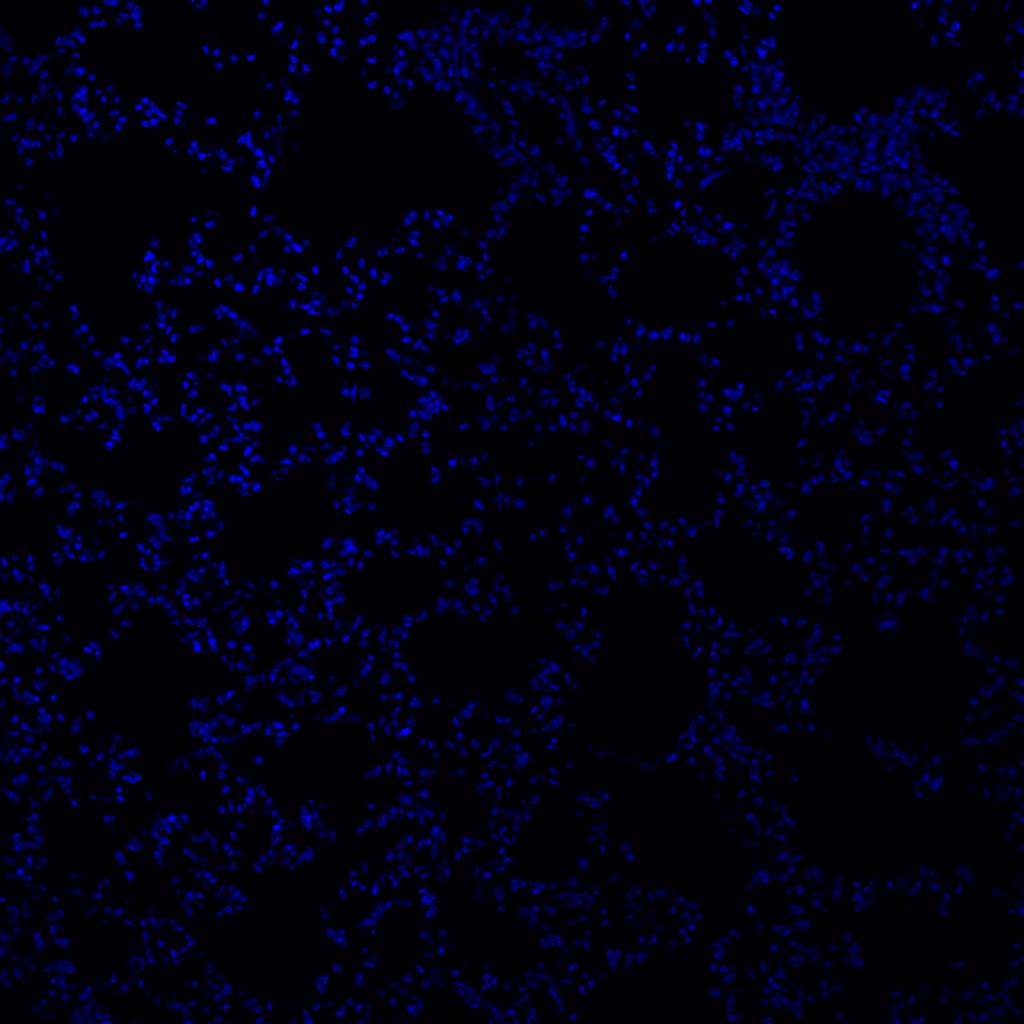

Supplement: Figure 6—source data 2. [file elife-88135-fig6-data2.zip › Figure6-source data2/Fig 6C left/y3-4_0004_C001T001.jpg]

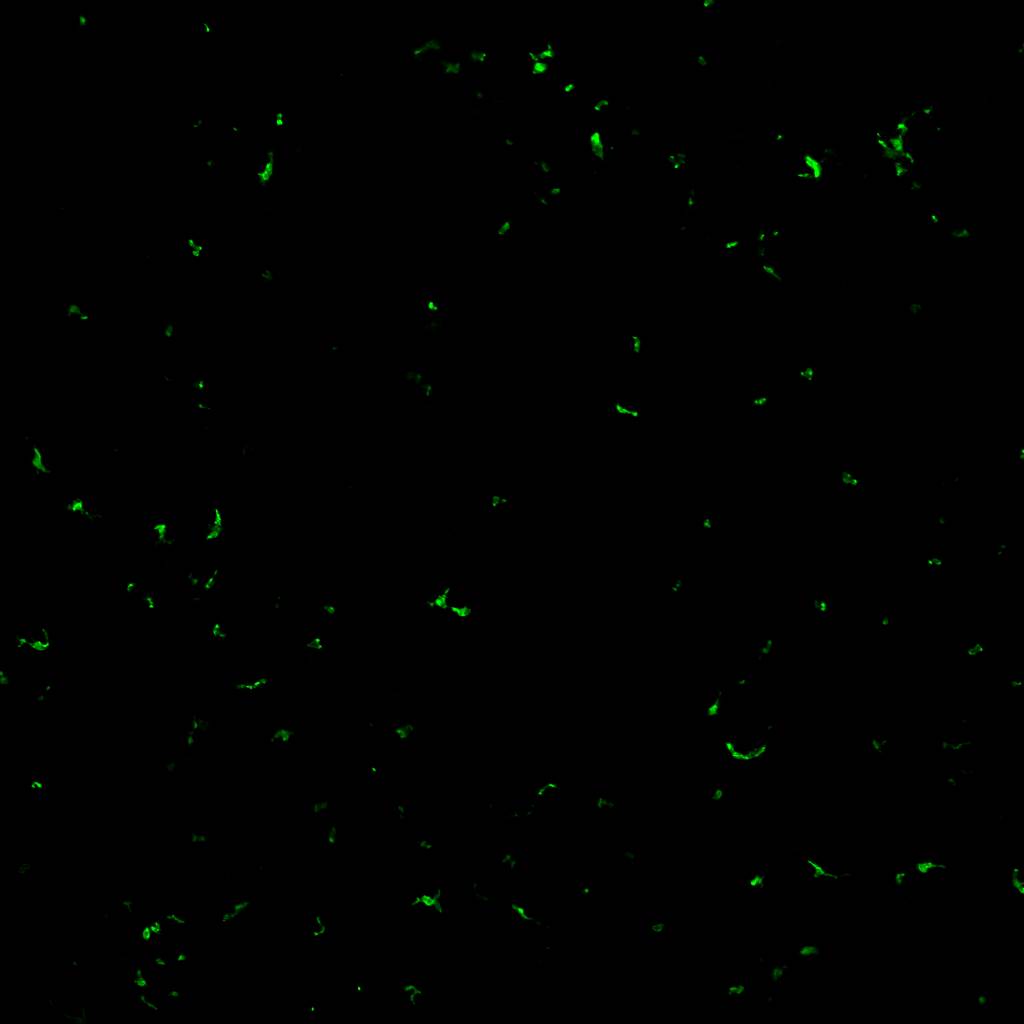

Supplement: Figure 6—source data 2. [file elife-88135-fig6-data2.zip › Figure6-source data2/Fig 6C left/y3-4_0004_C002T001.jpg]

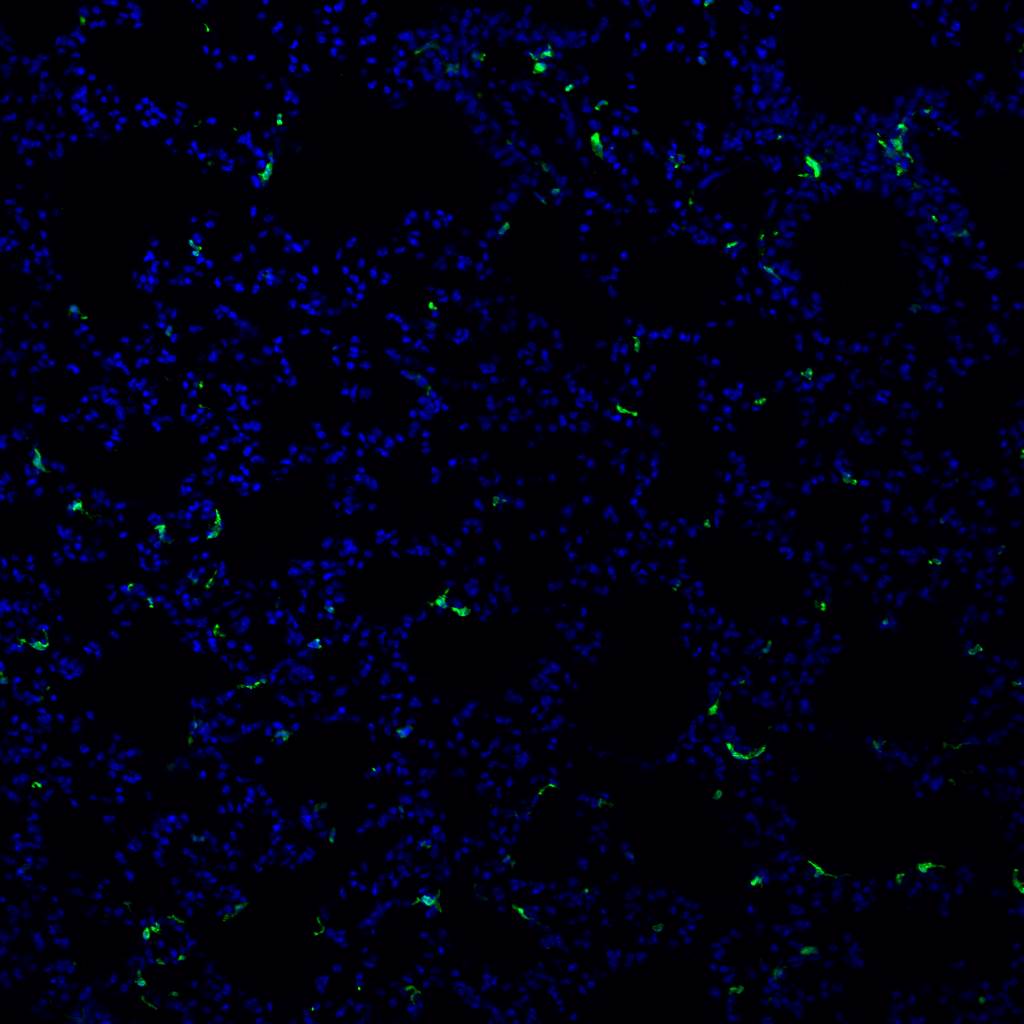

Supplement: Figure 6—source data 2. [file elife-88135-fig6-data2.zip › Figure6-source data2/Fig 6C left/y3-4_0004_T001.jpg]

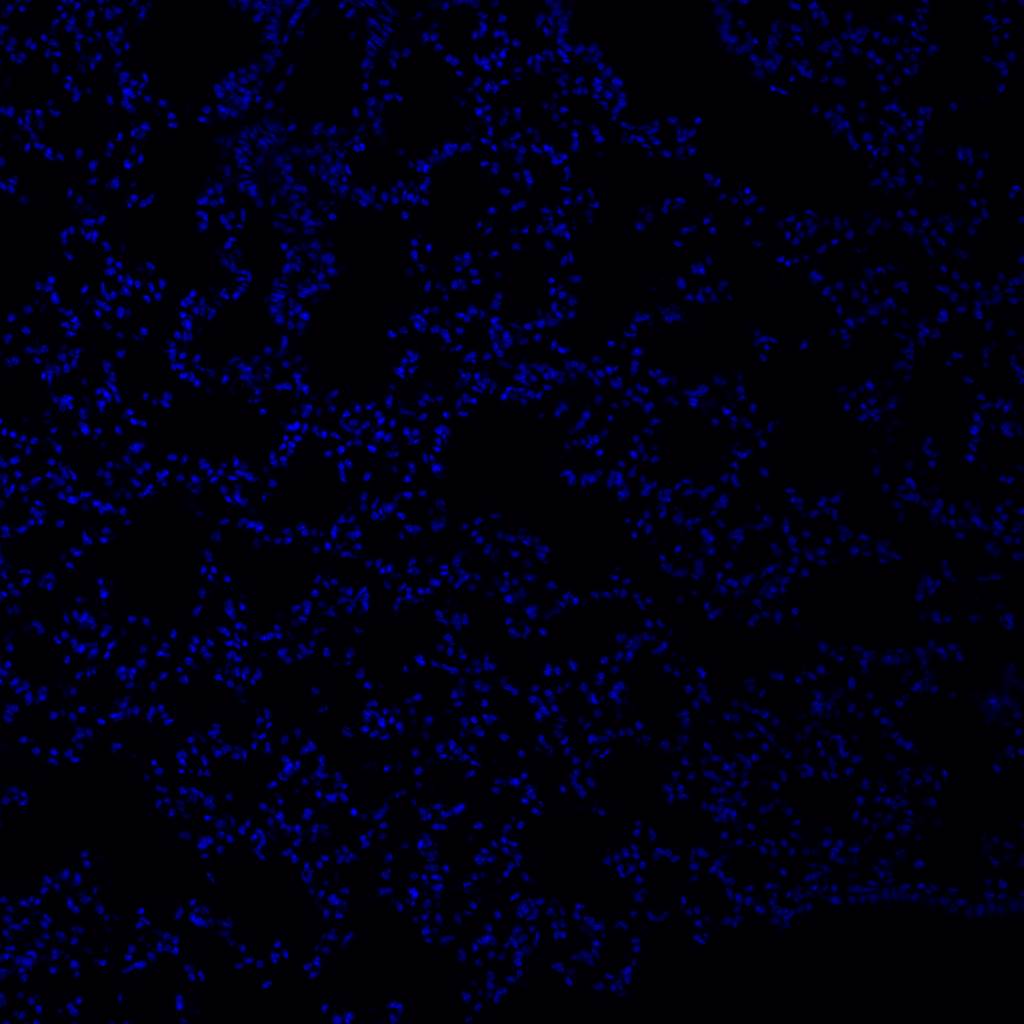

Supplement: Figure 6—source data 2. [file elife-88135-fig6-data2.zip › Figure6-source data2/Fig 6C middle/y2-1_C001T001.jpg]

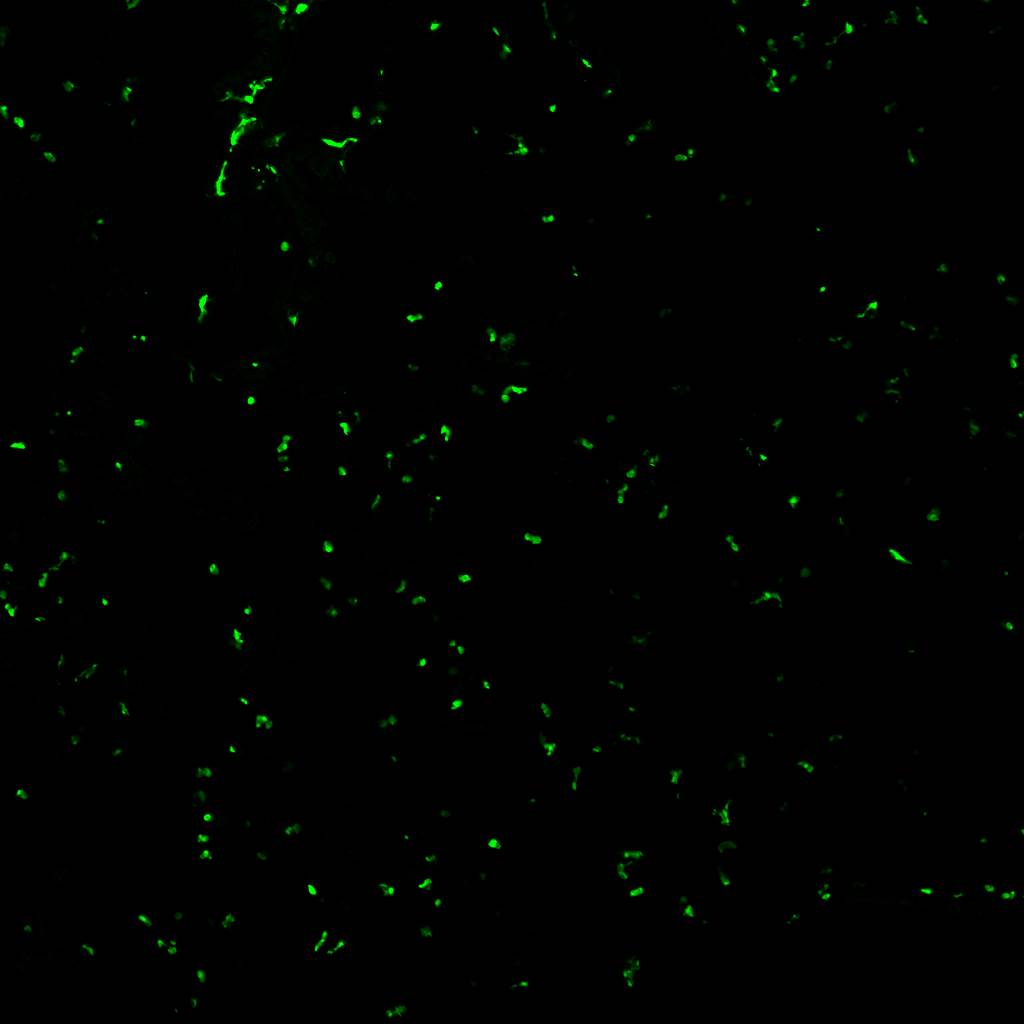

Supplement: Figure 6—source data 2. [file elife-88135-fig6-data2.zip › Figure6-source data2/Fig 6C middle/y2-1_C002T001.jpg]

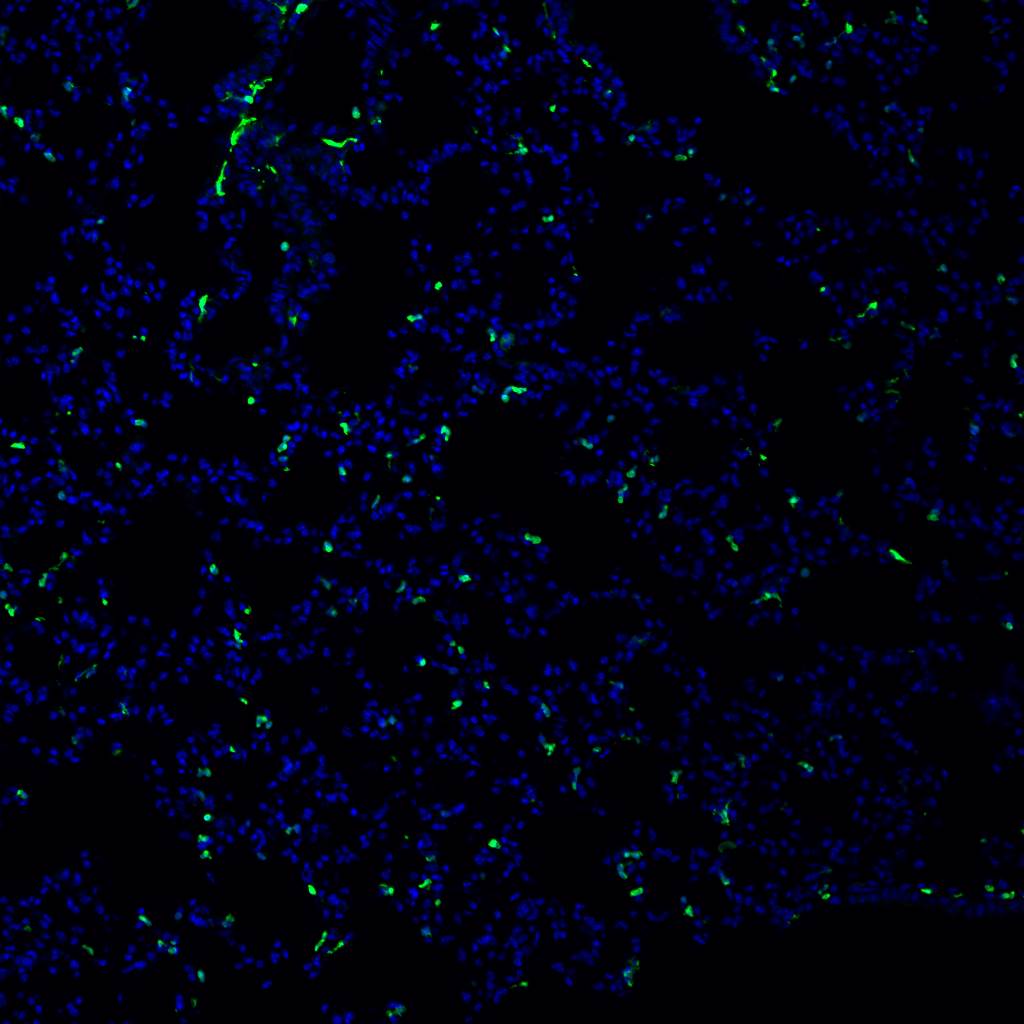

Supplement: Figure 6—source data 2. [file elife-88135-fig6-data2.zip › Figure6-source data2/Fig 6C middle/y2-1_T001.jpg]
